# ECG Classification Using an Optimal Temporal Convolutional Network for Remote Health Monitoring

**DOI:** 10.3390/s23031697

**Published:** 2023-02-03

**Authors:** Ali Rida Ismail, Slavisa Jovanovic, Naeem Ramzan, Hassan Rabah

**Affiliations:** 1 Institut Jean Lamour (UMR 7198), University of Lorraine, 54011 Nancy, France; 2 School of Computing, Engineering and Physical Sciences, University of the West of Scotland, Paisley PA1 2BE, UK

**Keywords:** ECG, temporal convolution, TCN, healthcare

## Abstract

Increased life expectancy in most countries is a result of continuous improvements at all levels, starting from medicine and public health services, environmental and personal hygiene to the use of the most advanced technologies by healthcare providers. Despite these significant improvements, especially at the technological level in the last few decades, the overall access to healthcare services and medical facilities worldwide is not equally distributed. Indeed, the end beneficiary of these most advanced healthcare services and technologies on a daily basis are mostly residents of big cities, whereas the residents of rural areas, even in developed countries, have major difficulties accessing even basic medical services. This may lead to huge deficiencies in timely medical advice and assistance and may even cause death in some cases. Remote healthcare is considered a serious candidate for facilitating access to health services for all; thus, by using the most advanced technologies, providing at the same time high quality diagnosis and ease of implementation and use. ECG analysis and related cardiac diagnosis techniques are the basic healthcare methods providing rapid insights in potential health issues through simple visualization and interpretation by clinicians or by automatic detection of potential cardiac anomalies. In this paper, we propose a novel machine learning (ML) architecture for the ECG classification regarding five heart diseases based on temporal convolution networks (TCN). The proposed design, which implements a dilated causal one-dimensional convolution on the input heartbeat signals, seems to be outperforming all existing ML methods with an accuracy of 96.12% and an F1 score of 84.13%, using a reduced number of parameters (10.2 K). Such results make the proposed TCN architecture a good candidate for low power consumption hardware platforms, and thus its potential use in low cost embedded devices for remote health monitoring.

## 1. Introduction

Electrocardiogram (ECG) is a rapid bedside inspection that measures the electrical activity generated by the heart as it contracts. It is commonly used to recognize diverse heart diseases such as arrhythmia, cardiomyopathy, coronary heart disease, cardiovascular disease, and many others. The inspection process has always been carried out by physicians and clinicians, which is a time-consuming procedure requiring significant medical and human resources to process the large amount of ECG data [1]. On the other hand, due to the diversity of ECG signals, many issues could arise, making this process of ECG inspection even more challenging. For example, the ECG of two healthy people may not be completely similar. Moreover, two patients suffering from the same heart disease could show different signs in their ECGs. Another issue could be that two different diseases have very close signals at the ECG level. It seems that there are no definite standards to be used in the diagnosis process [2]. For that reason, the use of artificial intelligence (AI) methods are needed, as these methods are learnable through accumulated experiences such that they could find hidden patterns that humans cannot find.

The computerized analysis of ECG signals was mainly meant to improve the diagnosis process, save time, and target rural and remote regions where medical specialists are not always affordable [3]. To this end, millions of ECG schemes are recorded worldwide every year, where most of them are automatically analyzed and decided afterwards. However, a false analysis is very likely, especially in the case of inexperienced clinicians who might endorse any automated results without further analysis. Such clinical mismanagement mostly ends up with a useless or even dangerous treatment. Thus, it becomes necessary that the ECG results are read and approved by well-experienced physicians. On the other hand, doctors highly recommend the modernization of existing computerized ECG analysis methods as well as the improvement of their robustness for more reliable medication.

Machine learning (ML) has proven to be eminently successful in different classification problems. This opened the door for its use in ECG analysis problems, and various ML-based methods have been recorded in this domain. In [4], ECG signals of normal people are collected and compared to ECG signals under tests using cross-correlation techniques. This allows for the detection of ECG signals of patients with myocardial infarction with an accuracy of 91.5% and a F1 score of 90.8%. In [5], a hybrid model of a decision tree with the C4.5 algorithm is applied on ECG features after they have been extracted using the genetic algorithm. The model was tested on the UCI arrhythmia dataset in two modes: 2-class and 16-class, ending up with highest evaluation metrics that have been recorded for the UCI arrhythmia dataset. In [6], two life-threatening arrhythmias, AFIB and AFL, are considered. A residual deep neural network architecture is proposed to detect the presence of such arrhythmias based on the RR interval of the ECG signals. The inputs are first extracted, denoised, and then normalized before being introduced to the network. A 10-fold cross-validation is carried out in training, leading to massive results in terms of accuracy and other metrics. In [7], the authors introduced a model based on a feed-forward multilayer neural network with error back propagation learning algorithm for the diagnosis of ischemic heart disease. The resulting high-order statistics facilitate the discrimination between the nonlinear dynamics of normal and diseased cases. Another feed-forward network for the same task was proposed in [8]. The network is deeper in terms of used layers, which makes it capable of classifying 6 ECG abnormalities that are representative of both rhythmic and morphological ECG abnormalities. The authors in this paper emphasize the need for expert review of borderline and complex cases after any automated classification. On the other hand, different prevalent deep learning architectures such as GoogLeNet, ResNet, and LSTM have shown great performance in the ECG domain [9,10,11]. This comes at the cost of storage and computation, as these models include millions of learnable parameters. Therefore, the adoption of such networks in hardware applications is often avoided. Other studies showing different cardiopathologies can also be considered in the future [12]. The main problem of machine learning methods that are data-driven remains the availability of datasets and different conditions of acquisition between different available datasets.

Convolution neural networks (CNN) are mainly intended for visual imagery analysis and computer vision tasks. However, with the great success these networks have shown in classification tasks, they started to be involved in automated ECG analysis as well. Nevertheless, the performance of convolutional networks could degrade due to the impurity of data as well as the imbalance in the number of examples between classes. For that, it is often required to utilize some effective data augmentation techniques with the raw data before the recruitment of robust-to-data convolutional network models. In this paper, we present an optimal architecture for sequential data processing based on 1D Temporal Convolutional Networks (TCN). A database of five classes named ECG5000 [13], originally established from the BIDMC Congestive Heart Failure Database [14], is considered in our study. The ECG database signals used are noise-free clean, which makes them ready for use without any preprocessing. Each sample includes a single heartbeat. The ECG5000 database is enhanced by three data augmentation techniques for better performance of the network. The network involves various diluted causal one-dimensional convolutions with padding. As a result, the output signal is the same length as the input heartbeat. The convolution layers are followed by a softmax unit that matches the heartbeats with their classes. Accordingly, the network is evaluated. Due to the unique internal design of TCNs, these networks are lighter in weight, faster, and more stable than conventional convolutional networks. This allows the implementation of efficient embedded systems suited to remote health monitoring systems. ECG analysis devices are thus realized using low complexity and power consumption hardware.

The rest of the paper is organized as follows: in Section 2, we provide a background on the machine learning methods used in ECG analysis. In Section 3, we present in detail our proposed temporal convolution network architecture; next we demonstrate the data augmentation techniques applied to the ECG data and designate the values used in the training process; and lastly we explain the standard used in the evaluation of the network. In Section 4, we display the results of the multiple trials that we carried out and compare our optimized model to some existing machine learning networks implemented on the same dataset. The whole paper is summarized and concluded in Section 5.

## 2. Background and Related Work

A large part of the world’s population resides in a spread-out remote or rural area. In these rural and remote areas, besides other basic needs of life, the overall access to medical facilities ranges from difficult to deficient, and the availability of doctors is scarce. The deficiency of timely medical advice and assistance to the patients, due to distance and lack of adequate infrastructure, is the source of critical situations and may lead to death in some cases. Remote health care is considered a serious candidate for facilitating access to health services for all. Sensing and actuating technologies along with big data analysis provide basic building blocks for remote health monitoring (RHM). The concept of RHM is not new, but newer and efficient systems are still being designed to overcome the weaknesses of existing systems, especially for rural areas. Indeed, in rural areas, the main challenges are related to communication latency and bandwidth availability, autonomy and energy consumption, and low cost devices.

**Figure 1:**
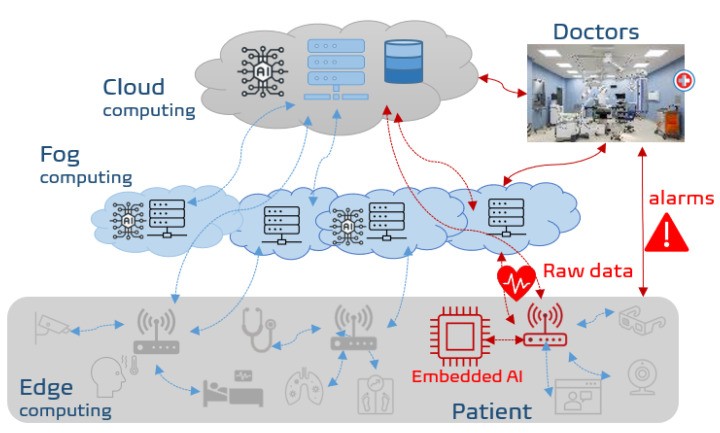
Overall framework of remote health monitoring in rural areas. In red is the flow related to ECG data. The classification is done by embedding intelligence near sensors and sending only alarms. If necessary, raw data can be sent to fog or cloud for further analysis or storage.

In the context of SAFE-RH (Sensing, ArtiFicial intelligence, and Edge networking towards Rural Health monitoring), a framework is proposed to cope with the above-mentioned problems by sending (and thus recording permanently) only the relevant data. Indeed, these relevant data to send are related to generation of alarms identified mostly by AI or machine learning (ML) driven methods, and thus significantly limit the bandwidth cost and communication overhead. Figure 1 shows the overall architecture of the proposed RHM system where ECG related flow is depicted in red.

**Figure 2:**
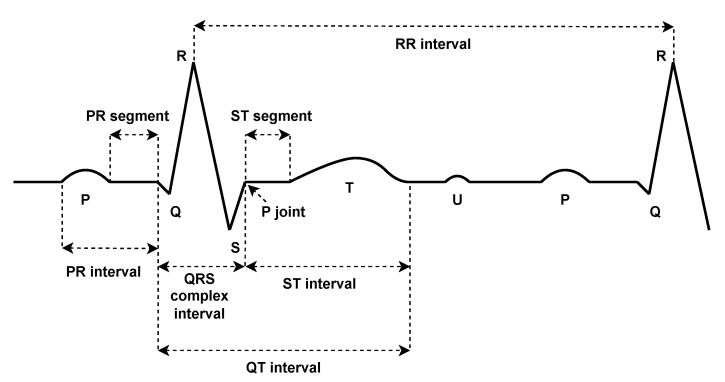
Normal ECG waveform.

The study of ECG signals has become an essential tool in the clinical diagnosis of various heart diseases. This study is mainly based on the detailed characteristics of the ECG signal. In detail, an ECG signal is composed of numerous heartbeats connected together. Each heartbeat consists of different parts, namely: P wave, QRS complex, and T wave (see Figure 2). A normal heartbeat is characterized by given amplitude values for its peaks (P, Q, R, S, T, and U), as well as given duration values for its intervals (PR, RR, QRS, ST, and QT) and segments (PR and ST). The variation of any of these values indicates a certain abnormality at the diagnosis level. More details of an ECG beat can be found in [15]. These peaks, intervals, and segments are called the ECG features, on which the ECG classification is mainly based. The ECG classification problem is often a multi-class classification problem. It includes several classes not limited to: normal (N), right bundle branch block (RBBB), and left bundle branch block (LBBB). An ECG classification process involves multiple steps starting from signal preprocessing, feature extraction, then normalization, and ending with classification. In the first phase, signals are filtered to remove any kind of possible noise that could affect the extraction of the features. This includes powerline interference [16], EMG noise [17], baseline wander [18], and electrode motion artifacts [19]. Various techniques are proposed for noise removal such as low and high pass linear phase filters. For baseline adjustment, techniques such as linear phase high pass filter, median filter, and mean median filter are usually employed. In the second phase, the main features are collected to be used as inputs to a classification model. The commonly used techniques for this purpose are: Continuous Wavelet Transform (CWT) [20], Discrete Wavelet Transform (DWT) [21], Discrete Fourier Transform (DFT) [22], Discrete Cosine Transform (DCT) [23], S-Transform (ST) [24], Principal Component Analysis (PCA) [25], Pan–Tompkins Algorithm [26], Daubechies Wavelet (Db4) [27], and Independent Component Analysis (ICA) [28]. For the normalization of the features, two main approaches are commonly used: Z-score [29] and Unity Standard Deviation (SD) [30]. Finally, in the classification stage, different models are utilized such as: Multilayer Perceptron Neural Network (MLPNN) [31], Quantum Neural Network (QNN) [32], Radial Basis Function Neural Network (RBFNN) [33], Fuzzy C-Means Clustering (FCM) [34], ID3 Decision Tree [35], Support Vector Machine (SVM) [36], Type2 Fuzzy Clustering Neural Network (T2FCNN) [37], and Probabilistic Neural Network (PNN) [38].

Different ECG classifications have been recorded. In [39], the authors established a four-class ECG classification problem using the RR intervals as inputs. The data were collected from the MIT-BIH arrhythmia database. The raw signals were first subjected to baseline adjustment. After that, the RR intervals were extracted using DWT and then normalized using Z-score. The classification was done using FCM with an accuracy of 99.05%. No other metrics were calculated. The same authors considered in [40] another four-class ECG classification problem using the same database but with different classes. The features used were the RR intervals and the R point location. The feature extraction was done using DWT with Daubechies wavelet of order 3. The outputs were classified at two stages: preclassification using FCM and final classification using a three-layer MLPNN. The final accuracy was up to 99.99%. A two-class problem was demonstrated in [41] using the MIT-BIH arrhythmia database. The RR interval and R location were extracted using Db4 discrete wavelet transform. An FFNN, trained with back propagation algorithm, was used as a classifier. The final results showed an accuracy of 95%, a sensitivity of 90%, and a specificity of 90%. Another two-class ECG classification problem was investigated in [42]. Data were collected from the two databases: MIT-BIH arrhythmia and normal sinus rhythm. Noise was removed by band pass filter. The features, RR interval and R peak, were first extracted using DWT and then normalized by zero mean. An FFNN, with error back propagation algorithm, was used for classification. The performance of the model was estimated by the calculation of the classification accuracy (96.77%) and Youden index (0.9415). In [43], the authors constructed an ECG classification model for six classes. Data were collected from the MIT-BIH arrhythmia database. The utilized features were QRSh (QRS complex height), QRS width, R peak, RRt interval (current RR interval at time t), and RRt+1 interval (next RR interval at time t+1). The Pan–Tompkins algorithm was used in feature extraction. A low pass linear phase filter was built for noise removal, whereas a median filter was built for baseline correction. Outputs were classified using a particle swarm optimization (PSO) RBFNN. The sensitivity and specificity of the model were 96.251% and 99.104%, respectively. In [44], an ECG classifier was built based on the MIT-BIH arrhythmia database for the classification of eight heart diseases. The involved features, R peak, QRS segment, and RR interval were normalized before being fed into the classification model consisting of a PNN (radial basis layer and competitive layer) and a three-layer FFNN with back propagation algorithm, using zero mean and unity standard deviation. The model evaluation showed a sensitivity of 98.508%, a specificity of 99.906%, and an overall accuracy of 98.710%. Another ECG classifier was investigated in [45] using the MIT-BIH arrhythmia database. R peak and RR interval were extracted by the use of DWT, whereas the classification process was done by an MLPNN. The model was shown to be reliable with a mean square error of 0.00621.

Convolution neural networks (CNN) have also been involved in ECG analysis. In [46], a 2D CNN approach for ECG classification is investigated. The sequential vectors representing the heartbeats are transformed into binary images via one-hot encoding [47] before being introduced to the network. The morphology of the heartbeats as well as the temporal relationship between every two adjacent heartbeats is captured in such images. The learning process is accelerated using ADADELTA [48], a per-dimension learning rate method for gradient descent. The network also involves a biased dropout [49] to mitigate the overfitting of the network. The network, when tested on the MIT-BIH arrhythmia database, has shown to be highly effective in the detection of various cardiovascular diseases. Another work is investigated in [50]. A 1D CNN approach for arrhythmia detection is proposed. The sequential data are extracted using two leads and then injected directly into the network without any preprocessing. Although the network achieves high accuracy when tested on the MIT-BIH database, some classes are hardly recognized. This can be the result of the impurity of the data as well as the imbalance between classes.

## 3. Method

### 3.1. Proposed Architecture

Until recently, sequential data was mostly analyzed and modelled using recurrence-dependent networks such as recurrent neural networks and LSTM architectures. However, the most problematic issue that arises in training such networks is the vanishing or exploding of gradients. In other words, the network is often incapable of learning its weights from long-past values. For that, convolutional neural networks, widely used for computer vision and visual imagery tasks, are currently used for signal processing as well, under the name 1D Temporal Convolutional Network (1D TCN). The convolution in a TCN is uni-dimensional, causal, and dilated. A causal convolution means that the computation at a given unit of the network only depends on present and past values; this suits sequential data where each point of a sequence depends on previous ones. On the other side, dilation is set to increase the sparsity of a kernel so that the receptive field of the convolution layer can be enlarged without using additional parameters. Note that a receptive field is the region in the input that produces a feature at the output. The receptive field (*R*) of a dilated convolution layer with factor *d* is R=d(k−1).

**Figure 3:**
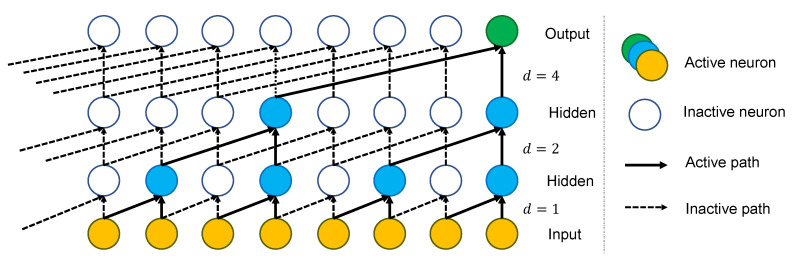
Dilated causal convolution in a TCN of four layers.

To build up a TCN, multiple convolution layers are stacked above each other as shown in Figure 3. The dilation factor of layer i≥1 is defined as $d_{i}=2^{i-1}$; this leads to an exponential growth of the receptive field size. Finally, with the scheme shown above, the receptive field of a TCN of *l* layers and convolution kernels of size *k* is defined as:

(1) $$R=2^{l}\left(k-1\right).$$

The convolution in a TCN layer is defined as follows:

(2) $$F\left(x\left(t\right)\right)=\left(x{⊛}_{d}f\right)\left(t\right)=\sum_{j=0}^{k}f\left(j\right)x\left(t-d\cdot j\right)$$

where *x* is the input sequence, *d* is the dilation factor, and *f* is a convolution filter of size *k* applied at time *t*. It should be noted that an input sequence of length *n* is introduced to the above vanilla 1D convolution layer in order to generate an output sequence of length n−k+1. A zero padding of length k−1 is often applied at the beginning of the sequence so that the length of the sequence is preserved.

**Figure 4:**
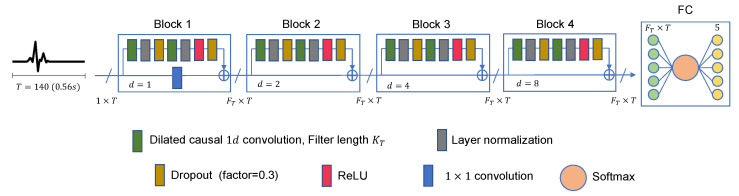
TCN architecture for ECG heartbeat classification. The input signal is made of 140 points (0.56 s). Number of filters is $F_{T}$. Filter length is $K_{T}$. Dilation factors of blocks are 1, 2, 4, and 8, respectively.

The full TCN model that we propose in this work is demonstrated in Figure 4. It is made up of multiple residual blocks, followed by a fully connected layer (FC), a softmax function, and a classifier. Each block contains a group of layers and a skip connection that links its input to its output. A 1×1 convolution is set on the skip connection of the first block in case the input and output mismatch in size. The skip connection is mainly used to revive gradients so that they can flow from one block to another without passing through non-linear activation functions. This, along with the dilation property of the utilized convolution, helps in solving the gradients’ exploding/fading issues. Within a residual block, the layers can be described as two sets connected in series where each set is composed of a dilated causal convolution layer, followed by layer normalization and spatial dropout layers. After the second normalization layer, a rectified linear unit (ReLU) is added. Note that the two convolution layers are identical (same filters and dilation factor) in one block, whereas they differ from one block to another. A normalization layer is added for faster and better performance of the TCN. It helps improve the stability of the network in case the weight’s initialization or the used regularization techniques are not helpful. This occurs by normalizing each of the inputs in the batch independently across all channels (features) using the mean and variance values [51]. This is different from batch normalization, mainly used in conventional convolutional networks, where normalization takes place at the level of batches in each channel separately. Layer normalization is well suited for sequence data where, unlike batch normalization, the batch size has no role. This allows the processing of large input sequences using any batch size for data division. Moreover, with layer normalization, parallelization is easily implemented with no need to have communication and synchronization between the different computing engines, as each one is computing separate data. On the other hand, in the dropout layer, some of the neurons are randomly deactivated during the network training. This aims to provide different forms of the network while training to avoid overfitting afterwards. Finally, the rectified linear unit (ReLU) performs a threshold operation on its input, where any element of a value less than zero is set to zero. This is done to achieve a non-linear transformation of the data so that they can be linearly separable during classification. The used values of the model hyperparameters can all be found in Section 4.

Temporal convolution networks have been shown to outperform recurrent neural networks [52]. In terms of memory, the sparse kernels in TCNs allow the prediction of a time series from their long-past values using a very low number of parameters. In recurrent networks, this is done by the use of cycles and condensed recurrent connections, resulting in a large number of parameters. Moreover, the internal structure of a TCN is independent of the input signal. This allows the processing of excessively long sequences using a small TCN structures. Additionally, the receptive field size in such networks is easily tuned by modifying the number of layers, the filter size, and the dilation factors; this facilitates control of the model’s memory for various requirements. In terms of performance, temporal convolution networks are much faster than recurrent networks. This is due the fact that these networks compute their outputs in parallel. On the other side, the structure of a TCN leads to more stable gradients where these gradients vary in the direction of the layers not in the temporal direction (also thanks to the residual connections). Temporal convolution networks are not without flaws. Indeed, these networks do not function well in the case of domain transfer especially from a domain that requires a short history to another that requires a long one.

### 3.2. Data Augmentation

**Figure 5:**
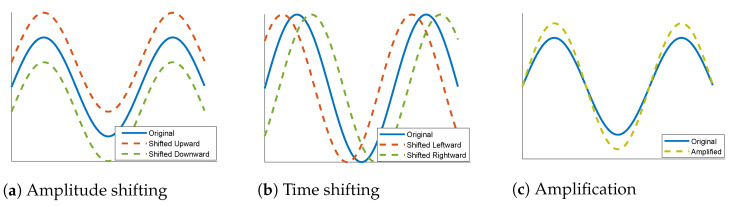
Data augmentation techniques applied to the classes PVC, SP, and UB of the ECG5000 dataset.

In terms of classification problems, the lack of sufficient training samples of certain classes is often fixed by using data augmentation techniques. This occurs by adding new copies of the existing samples of the deficient classes after applying certain minor alterations or using machine learning models to generate new examples in the latent space of the original data. Various techniques can be found in the literature for the augmentation of image data such as rotation, flipping, cropping, and color transformation [53]. Such mechanisms preserve the main features of an image while providing a bigger space for training. This seems more complicated in the case of one-dimensional data. In this work, we propose three simple types of data augmentation that can be applied to the ECG signals: amplitude shifting, time shifting, and amplification. Amplitude shifting, as shown in Figure 5a, means moving the signal a certain number of steps either upward or downward. Time shifting is presented in Figure 5b; it is about moving the signal a given number of steps either to the right or to the left. Amplification, as shown in Figure 5c, is done by vertically extending the signal by a certain ratio. Like conventional 2D data augmentation techniques, these proposed techniques, along with many others, provide additional data for use in training while preserving the main features of a signal.

### 3.3. Training Process

The general TCN model, proposed in Section 3.1 and shown in Figure 4, is trained using Adam optimizer on batches of size 20 at a learning rate of 0.0025. Different trials are carried out, as will be shown in Section 4, by changing the number of blocks, the number of filters, the filters’ size, and the applied data augmentation techniques. The training process lasts for 250 epochs. The dropout ratio of all dropout layers is 0.3. The raw dataset (before augmentation) is divided into two sets: training dataset and testing dataset, with a ratio of 90:10. The training dataset is shuffled once before use, and no cross-validation is applied. On the other hand, the evaluation of the network is done on the testing dataset at the end of every epoch. The final accuracy is chosen at the epoch where the parameters of the network produce the minimum loss.

### 3.4. Network Evaluation

Classification model performance is mostly evaluated based on the “confusion matrix” [54]. This is a quite common measure that can be applied to both binary and multiclass classification problems where the counts of predicted and actual outcomes are all represented. For each class, four quantities can be identified: TP, FP, TN, and FN. The term “TP” denotes True Positive, which represents the number of positive examples that are correctly classified by the model. Similarly, the term “TN” stands for True Negative, which represents the number of negative examples that are correctly classified. The term “FP” stands for False Positive, which is the number of negative examples classified by the model as positive; whereas the term “FN” denotes False Negative, i.e., the number of positive examples classified as negative. The most commonly used criterion in the evaluation of a classification model is accuracy, which presents the fraction of true examples over all examples:

(3) $$Accuracy=\frac{All\;correct}{All}=\frac{TP+TN}{TP+TN+FP+FN}.$$

However, the accuracy defined in Equation (3) can be misleading when the datasets used for training and test purposes are imbalanced. For that, there are other metrics that could be involved in the evaluation process for better analysis: precision and sensitivity. Precision is the proportion of correctly classified positive cases, i.e., the fraction of positive examples over the total predicted positive examples. On other hand, sensitivity is the proportion of correctly recognized actual positive cases, i.e., the fraction of positive instances over the total actual positive instances. The formulas are defined below:

$$\begin{array}{cc} \; & Precision=\frac{True\;Positives}{Predicted\;Positives}=\frac{TP}{TP+FP};\\ \; & Sensitivity=\frac{True\;Positives}{All\;Actual\;Positives}=\frac{TP}{TP+FN}. \end{array}$$

It should be noted that the two quantities above are computed for each class separately, and therefore the overall quantities are deduced by averaging. For the sake of brevity, precision and sensitivity can be combined into one term, the F-score, as follows:

(4) $$F_{\beta }=\left(1+\beta^{2}\right)\frac{Sensitivity\times Precision}{(\beta^{2}\cdot Sensitivity)+Precision}.$$

The balanced F-score is the harmonic mean of precision and recall; that is, the $F_{1}\text{-}score$ (β=1):

(5) $$F_{1}=2\cdot \frac{Sensitivity\times Precision}{Sensitivity+Precision}.$$

It is always preferable to achieve a good performance of the TCN model using the most concise network structure. For that, the number of parameters that comprise the TCN is another criterion to be considered in the evaluation process. A TCN, as shown in Figure 4, is made up of multiple layers, where each distinct layer involves a different number of parameters. The only layers that have no learnable parameters are the input layer, the ReLU layer, and the dropout layer. The input layer only provides the shape of the input signal and has nothing to do with the training process. The ReLU performs a threshold operation on its input *x* according to the fixed equation f(x)=max(0,x). Similarly, this layer is not changed during the training phase. The dropout layer is set to reduce the number of activated neurons in the training phase where elimination takes place in a merely random way. The layers that include learnable parameters are the convolution layer and the normalization layer. Each convolution layer has two types of parameters: weights and biases. The weights are learned during the training process. They are matrices that affect the prediction ability of the model, which is altered during the back-propagation process based on the used optimization strategy. The biases are set to delay or accelerate the activation of nodes. The total number of parameters in a convolution layer is the sum of all present weights and biases. Knowing that the size of a filter of a given convolution layer is denoted by $K_{T}$, the number of filters of this layer is denoted by $F_{T}$, and the number of filters of the previous layer is denoted by $F_{T}^{p}$, then the total number of parameters of this convolution layer ($P_{conv}$) is defined as follows:

(6) $$P_{conv}=W+B=\left(K_{T}\times F_{T}^{p}\times F_{T}\right)+F_{T}$$

where *W* and *B* are the number of weights and the number of biases of the convolution layer, respectively. Note that the dilation, stride, and padding are hyperparameters that do not interfere in the learning process [55,56]. Similarly, a normalization layer also has two learnable parameters of its own: offset (also called beta) and scale (gamma). Each channel of a normalization layer has one parameter of each kind. Attached to the convolution layer, the normalization layer thus has $F_{T}$ betas and $F_{T}$ gammas. Hence, the total number of parameters in a normalization layer is:

(7) $$P_{norm}=2\times F_{T}.$$

## 4. Results and Discussion

The TCN model that we proposed in Section 3.1 is tested on the ECG5000 dataset [13] that has been collected from the BIDMC Congestive Heart Failure Database [14]. The raw record is composed of 17,998,834 data points including 92,584 heartbeats. The heartbeats are first extracted and then interpolated so that they all become the same length. After that, the heartbeats are annotated according to five classes holding the following labels: Normal (N), R-on-T Premature Ventricular Contraction (Ron-T PVC), Premature Ventricular Contraction (PVC), Supraventricular Premature or Ectopic Beat (SP or EB), and Unclassified Beat (UB). About 128,570 annotated heartbeats are present from which a dataset of 5000 randomly selected heartbeats is created. The new dataset is then divided into two sets: one for training that contains 4500 samples and another for testing that contains 500 samples.

On the other hand, different forms of the TCN model are built and experimentally tested. All details are summarized in Table 1, where 12 different experiments are demonstrated. The number of blocks varies between 3 and 5. The size of the filters of the dilated convolution layers is constant in one block whereas it grows from one block to another. However, the number of filters is always the same. Consequently, the number of parameters of the TCN model is calculated (see Section 3.4 for more details). In all experiments, the mini-batch has a size of 20, whereas the training process lasts for 250 epochs. The dilation factors ($d_{i}$) of the five blocks (if they exist) are 1, 2, 4, 8, and 16, respectively. For data augmentation, different factors are tried out on the three classes: PVC, SP, and UB. In all experiments, the classes PVC and UB (classes 3 and 5, respectively) are augmented 24 times. On the other hand, class SP is augmented 12 times in Experiments 1–10, 24 times in Experiment 11, and 6 times in Experiment 12. Based on the available training data and the size of the training batch, the size of one epoch is determined. The best results are obtained for Experiments 1 and 5. In Experiment 1, the TCN model involves about 10,200 parameters and achieves an F1 score of 84.13% and an accuracy of 96.12%. The TCN model in Experiment 5 involves about 39,900 parameters and achieves an F1 score of 85.43% and an accuracy of 96.62%.

**Figure 6:**
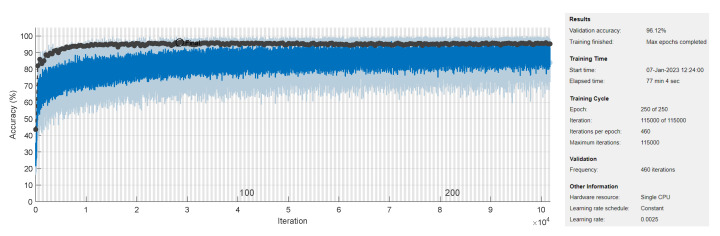
Training and validation process of TCN model in Experiment 1 of Table 4.

**Figure 7:**
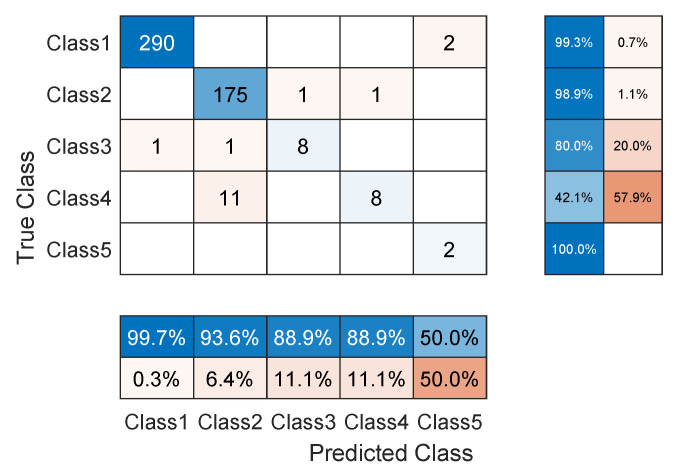
Confusion matrix of the TCN model proposed in Experiment 1 of Table 1, applied to the ECG5000 dataset.

**Figure 8:**
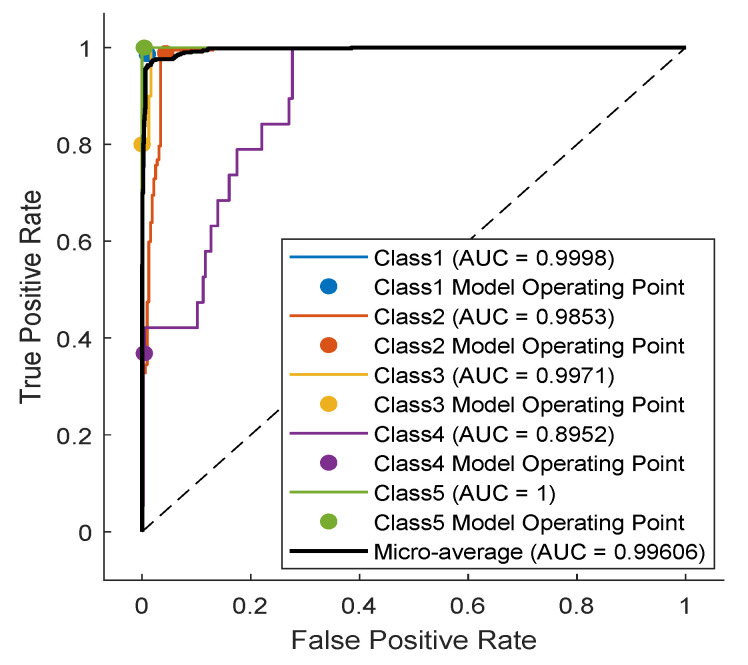
ROC curves of the trained model with marked operating points (dots) obtained for the five-class use case (Experiment #1, see Table 1).

The TCN model in Experiment 1 is the most optimal among all models present in Table 1. In detail, it has convolution layers that involve 16 filters ($F_{T}=16$). The size of the filters augments from one block to another as $K_{T}\in \left\{2,4,6,8\right\}$. The training data of classes PVC and UB are augmented 24 times: 4 times by amplitude shifting, 12 times by time shifting, and 8 times by amplification; whereas the training data of class SP are augmented only 12 times: 2 times by amplitude shifting, 6 times by time shifting, and 4 times by amplification. More details are found in Table 2 and Table 3. After data augmentation, the number of samples of the training data set grows from 4500 to 9204. The mini-batch has a size of 20, which leads to the distribution of the samples on an epoch of 460 iterations. With 250 epochs, the training process ends after 115,000 iterations. The evolution of the training and testing accuracy is shown in Figure 6. As shown, the model gains its optimal parameters after nearly 75 epochs with a testing accuracy of 96.12%. A more detailed evaluation of Experiment 1 can be done based on the confusion matrix shown in Figure 7. The sensitivity percentages of the classes are presented to the right of the 5×5 confusion matrix, whereas the precision percentages are placed below it (all in blue). The classification results for Classes 1–3 are very competitive where the sensitivity and precision percentages are high. By contrast, the classification is misleading for Class 4 with a sensitivity of 42.1%, and for Class 5 with a precision of 50%. In the latter two classes, the applied data augmentation techniques improve the classification results to a limited range. We should mention here that without data augmentation, the sensitivity of Class 4 is 31.5%, whereas the precision of Class 5 is 25%. Apart from data augmentation, multiple techniques could be employed to handle imbalanced data such as K-fold cross-validation, the use of specialized models like XGBoost, and the aggregation of more raw data. The receiver operating characteristic (ROC) curves of the five classes are shown in Figure 8, which demonstrates an excellent classification of the proposed model for four classes (Class 1, Class 2, Class 3, Class 5) and satisfactory classification results for Class 4.

The ECG5000 dataset has been adopted in various classification works for heart disease detection. The comparison between our optimal model and some state-of-the-art models is demonstrated in Table 4. We show in this table the accuracy, the F1 score (combination between sensitivity and precision), and the number of parameters of the different models. As shown, our TCN model with the fewest number of parameters among all other existing models (10.2 K) attains the highest accuracy among all of these models (96.12%). On the other hand, our TCN model affords an F1 score of 84.13%, which is lower only than that of the TCN model proposed in [57]. The effect of the applied data augmentation is also investigated. The accuracy is only 93.4% and the F1 score is down to 70.18% for the same TCN model when applied to the raw data.

**Figure 9:**
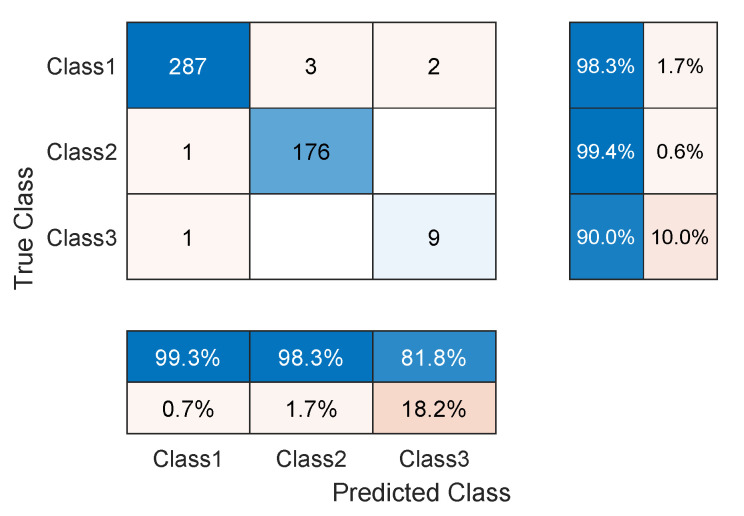
Confusion matrix of the TCN model proposed in Experiment 1 of Table 1, applied to the ECG5000 dataset excluding classes SP and UB.

**Figure 10:**
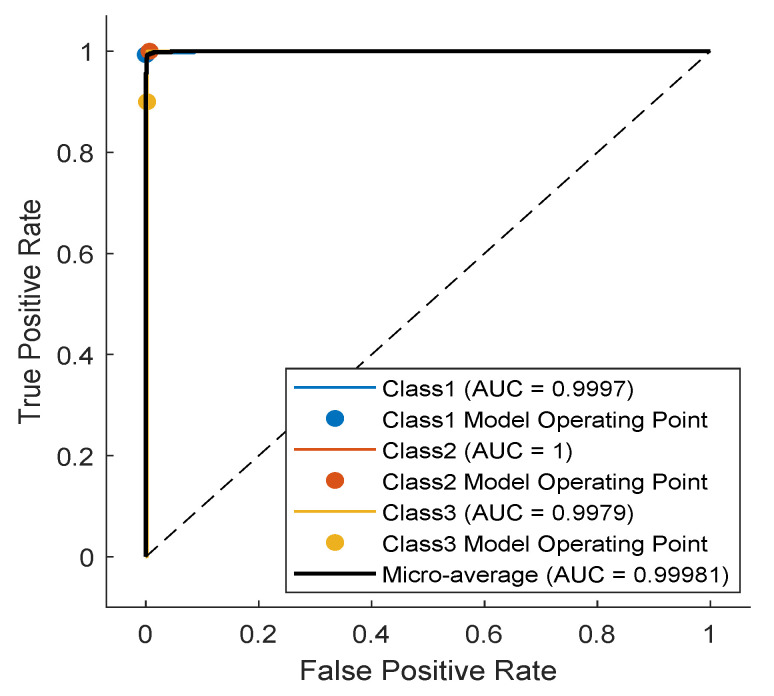
ROC curves of the trained model corresponding to Experiment 1 of Table 1, where the network is trained only on the first three classes.

As noticed in the confusion matrix of Figure 7, the slight deficiency in the F1 score is mainly due to classes SP and UB (see orange boxes). For that, we retrain the network while excluding these classes. As shown in Table 4, the accuracy now is up to 98.54%, whereas the F1 score reaches 94.51%. The detailed results are present in the confusion matrix of Figure 9. The receiver operating characteristic (ROC) curves of the three classes are shown in Figure 10 with an AUC≈1 demonstrating excellent classification results for this use case.

## 5. Conclusions

In this paper, we proposed a 1D Temporal Convolutional Network (TCN) based architecture for ECG classification of five heart diseases. The main goal was to provide a low complexity architecture aimed at being used in low-cost embedded devices for remote health monitoring. The proposed architecture is characterized by its simplicity and the lowest number of used parameters compared to the state-of-the-art approaches (10.2 K parameters < 15 K in the literature). Moreover, the proposed architecture is outperforming all existing state-of-the-art ML methods in terms of overall accuracy and F1 score, reaching up to 96.12% and 84.13%, respectively. The ROC curves of the proposed model show excellent classification performances with an average AUC≈1. As future work and perspectives, an extensive study to interpret and explain the obtained results will be conducted, along with the study of quantization and pruning of the network’s parameters and their influence on the overall accuracy of the proposed architecture as well as the hardware resources used and power consumption needed. Both microcontroller-based and circuit-specific implementations will be targeted with a comparison in terms of power consumption, ease of implementation and use, and overall cost.

## Figures and Tables

**Table 1:** Different forms of the TCN model proposed in Section 3.1 with different data augmentation cases. #Epochs=250 and Mini-BatchSize=20.

Exp	#Blocks	#Filters	Filter Size	#Parms	Data Augmentation Factor	Epoch Size	F1 Score	Accuracy
#1	4	16	{2,4,6,8}	10.2 K	class3:24, class4:12, class5:24	460	84.13%	96.12%
#2	3	16	{2,4,6}	6 K	class3:24, class4:12, class5:24	460	68.54%	94.80%
#3	5	16	{2,4,6}	15.4 K	class3:24, class4:12, class5:24	460	83.76%	96.26%
#4	4	8	{2,4,6,8}	2.7 K	class3:24, class4:12, class5:24	460	73.40%	95.20%
#5	4	32	{2,4,6,8}	39.9 K	class3:24, class4:12, class5:24	460	85.43%	96.62%
#6	4	16	{4,6,8,12}	14.9 K	class3:24, class4:12, class5:24	460	81.04%	95.87%
#7	4	32	{4,6,8,12}	58.4 K	class3:24, class4:12, class5:24	460	75.60%	96.07%
#8	3	16	{4,6,8,12}	8.6 K	class3:24, class4:12, class5:24	460	78.32%	95.57%
#9	3	16	{6,8,12,14}	12.2 K	class3:24, class4:12, class5:24	460	73.57%	95.70%
#10	3	32	{2,4,6}	23.3 K	class3:24, class4:12, class5:24	460	73.80%	96.00%
#11	4	16	{2,4,6,8}	10.2 K	class3:24, class4:24, class5:24	566	80.85%	96.35%
#12	4	16	{2,4,6,8}	10.2 K	class3:24, class4:06, class5:24	407	79.70%	96.02%

**Table 2:** Data augmentation applied on ECG5000 dataset in Experiments 1–10 of Table 1.

Class	Data Augmentation Type		
	Amplitude Shift	Time Shift	Amplification
PVC	${\left[-0.4,0.4\right]}_{0.2}$	${\left[-6,6\right]}_{1}$	${\left[1.05,1.4\right]}_{0.05}$
SP	${\left[-0.4,0.4\right]}_{0.4}$	${\left[-5,5\right]}_{1}$	${\left[1.05,1.2\right]}_{0.05}$
UB	${\left[-0.4,0.4\right]}_{0.2}$	${\left[-6,6\right]}_{1}$	${\left[1.05,1.4\right]}_{0.05}$

**Table 3:** Distribution of samples of ECG5000 dataset in Experiments 1–10 of Table 1, before and after data augmentation.

Class	Count				
	Testing Set	Before Augmentation		After Augmentation	
		Training Set	Total	Training Set	Total
N	292	2627	2919	2627	2919
Ron-T PVC	177	1590	1767	1590	1767
PVC	10	86	96	2150	2160
SP	19	175	194	2287	2306
UB	20	2	24	550	552
Total	500	4500	5000	9204	9704

**Table 4:** Evaluation of different methods on the ECG5000 dataset.

Architecture	Accuracy (%)	F1 Score (%)	#Parameters
TCN [57]	94.2	89.0	14.88 K
LSTM-FCN [58]	94.1	72.5	404.74 K
CCN [58]	93.4	81.5	266.37 K
LSTM [58]	93.1	68.9	138.37 K
1-NN (L2 dist.) [59]	92.5	54.9	70 K
Our TCN	96.12	84.13	10.2 K
Our TCN (First 3 classes)	98.54	94.51	10.2 K
Our TCN (Without Data Augmentation)	93.4	70.18	10.2 K

## Data Availability

http://www.timeseriesclassification.com/description.php?Dataset=ECG5000.
